# Effect of handholding on heart rate variability in both patients with cancer and their family caregivers: a randomized crossover study

**DOI:** 10.1186/s13030-021-00217-y

**Published:** 2021-09-23

**Authors:** Hiroko Sakuma, Hideaki Hasuo, Mikihiko Fukunaga

**Affiliations:** Department of Psychosomatic Medicine, Kansai Medical University, Shinmachi 2-5-1, Osaka 573-1090 Hirakata, Japan

**Keywords:** Handholding, Family caregivers, Heart rate variability, Self-care, Autonomic functions

## Abstract

**Background:**

Many family caregivers of patients with cancer feel guilty about self-care. A meaningful relationship with patients reduces such negative feelings and functions as self-care for family caregivers. Moreover, handholding improves autonomic functions in non-cancer patients. However, the effects of handholding on both patients with cancer and family caregivers remain unknown.

**Methods:**

We evaluated the effects of handholding on heart rate variability (HRV) in patients with cancer and their family caregivers. This randomized crossover study divided patients with cancer and their family caregivers into two trial groups: Handholding trial (the family caregiver holds the patient’s hand for five minutes) and Beside trial (the family caregiver stays beside the patient without holding their hand). The study included 37 pairs of patients with cancer who received treatment in the cancer department of a university hospital in Japan and their family caregivers (n = 74). The primary end-point was the change in HRV before and during the intervention.

**Results:**

The median performance status of the patients was 3. An interaction was observed between trials in the standard deviation of the normal-to-normal interval (SDNN) of HRV for family caregivers (F = 7.669; *p* = 0.006), and a significant difference in time course was observed between the trials (before *p* = 0.351; during *p* = 0.003). No interaction was observed between trials in the SDNN for patients (F = 0.331; *p* = 0.566). Only a main effect in time course (F = 6.254; *p* = 0.014) was observed. SDNN increased significantly during the intervention in both trials (Handholding trial: *p* = 0.002, Beside trial: *p* = 0.049).

**Conclusions:**

Handholding improves autonomic functions of family caregivers and may function as self-care for family caregivers.

**Trial registration:**

UMIN000020557. Registered on January 15, 2016.

## Background

Family caregivers are less motivated to engage in self-care because they feel guilty about not effectively being involved in patient care or of taking care of themselves [1]. For example, in a study that introduced mindfulness-based stress reduction to family caregivers of lung cancer patients, the family caregivers prioritized the patients’ well-being, and their distress was not reduced [2]. Family caregivers who do not spend time engaging in self-care because of their sense of responsibility for patient care feel a greater burden [3], which results in unsuccessful psychological and emotional management for themselves [4]. Previous literature has suggested that there is an association between a heavy patient care burden and an increased mortality rate for family caregivers [5].

The importance of self-care support for both family caregivers and patients with cancer has been recognized [6]. There are two types of self-care support. One type is direct intervention, which directly introduces self-care techniques such as relaxation. Several studies have reported that educating family caregivers about the benefits of relaxation is effective in improving their self-care practice [7, 8]. The other type is indirect intervention, which provides skill training for family caregivers to become better involved in patient care [9]. Moreover, effective interaction with patients has been reported as a form of self-care for family caregivers [1].

A family caregiver holding a patient’s hand is one of the most common actions in daily life. Many previous studies suggest the usefulness of touching or massage as complementary and alternative forms of medicine [10, 11]. However, to our knowledge, only two studies have evaluated the effectiveness of handholding by family caregivers. One study evaluated the effects of handholding by family caregivers on gastric motor function using extracorporeal ultrasound in patients with cognitive impairment [12]. The other study evaluated the gastric motor function of patients with decreased levels of consciousness using extracorporeal ultrasound while family caregivers were holding their hand in a relaxed state [13]. In both studies, the patients’ gastric motor function and autonomic function were significantly increased during handholding. However, the previous studies were limited by the high invasiveness of the gastric motor function measurement with extracorporeal ultrasound and the lack of evaluation of autonomic function in family caregivers. A study using a less invasive procedure for measuring autonomic function and evaluating the effects on both patients and family caregivers is necessary. Heart rate variability (HRV) is an indicator of autonomic function that can be measured less invasively and simultaneously in both patients and family caregivers.

This study aimed to evaluate the effect of family caregivers holding the hands of their family members with incurable cancer (patient) by measuring the HRV of both caregivers and patients.

We hypothesized that handholding serves as a direct intervention for family caregivers’ self-care support by improving the HRV of family caregivers and as an indirect intervention by improving the HRV of patients.

## Methods

### Study participants

In this study, we defined family caregivers as family members who directly provided care to a relative with cancer including spouses; patients were defined as incurable cancer patients with a performance status (PS) of 2 or higher. This study was conducted from January 2018 to July 2020 at Kansai Medical University Hospital in Japan. The exclusion criteria were as follows: (1) paralytic symptoms of bilateral upper limb desensitization levels and (2) mental disorders such as cognitive impairment that prevented the patient from being able to communicate.

### Study design and schedule

**Fig. 1:**
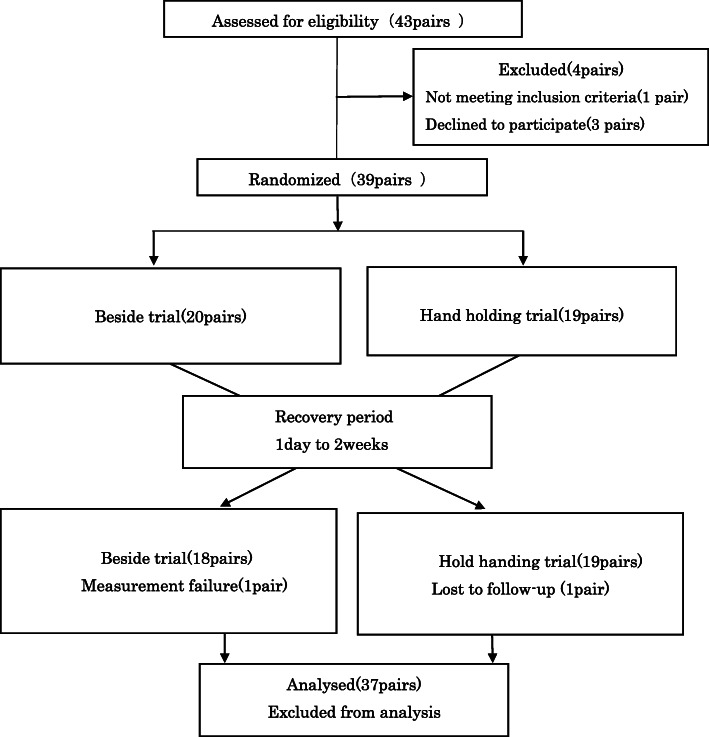
The study flow chart

We conducted this randomized crossover trial in pairs of family caregivers and patients with cancer. The patients were allocated into either the Handholding trial, in which the family caregiver held the patient’s hand for 5 min, or the Beside trial, in which the family caregiver remained beside the patient without holding their hand. Allocation was performed according to a computer-generated algorithm using minimization methods, with a 1:1 allocation ratio. The allocation management was performed by one doctor who is also a research collaborator. He had no direct contact with the participants. Figure 1 shows the study flow chart.

**Fig. 2:**
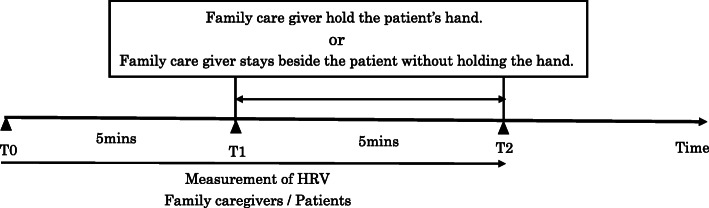
The study schedule

We connected the HRV components (mybeat WHS-2; Union Tool. Co.,) to a special electrode pad attached directly to the participant’s chest. Their HRV waveform was displayed on the screen of a tablet personal computer (iPad mini; Apple, Cupertino CA, USA) in real time. We measured HRV among the participants before administering the actions. Figure 2 shows the study schedule. Because we prioritized the family caregiver’s usual style, the detail and positioning of handholding were not specified.

### Outcomes

The primary outcome was the amount of change in HRV before and during the intervention.

### Measurements

#### Heat rate variability

HRV, which is the function of the heartbeat interval measured from electrocardiogram or pulse waves, is used to reflect autonomic nerve activity. We estimated the standard deviation of the normal-to-normal interval (SDNN), the low frequency (LF; 0.04–0.15 Hz) component, and the high frequency (HF; 0.15–0.4 Hz) component.

SDNN, the standard deviation of the R-R interval in an electrocardiogram, is obtained through a time domain analysis. SDNN indicates overall fluctuations of the R-R intervals. LF and HF components are recorded throughout several minutes of measuring HRV. These components are obtained by domain analysis. LF reflects both sympathetic and parasympathetic activities and HF reflects parasympathetic activity [14–17].

The mean values for resting HRV in adults are as follows: SDNN = 50 ms, LF = 519 ms^2^, and HF = 657 ms^2^ [18].

#### Sample size calculation

In a previous study that examined the effect of resonant breathing in family caregivers of cancer patients [7], the difference in the mean SDNN during the five-minute intervention between the intervention trial and the control group was 11.5 ms and the standard deviation was 15.2 ms. Based on those results, with a two-sided significance level of 5 % and a statistical power of 0.8, 28 participants were required for each group in this study. Considering a dropout rate of 20 %, the sample size was set at 35.

### Statistical analysis

Data were reported as the mean with SD, as appropriate.

To conduct comparisons between groups, we used time course as the within-subject factor and group as a between-subject factor in a two-way repeated measures analysis of variance. When interactions were observed in the two-way analysis of variance (ANOVA), subtests were performed using the Bonferroni post-hoc test. Changes in the time course of SDNN, LF, and HF scores (before–during) were analyzed using the paired t-test for each group.

A *p*-value less than 0.05 was considered statistically significant. The statistical analyses were performed using SPSS version 18.0 J for Windows (SPSS, Inc. IBM, Chicago, IL).

## Results

Tables 1 and 2 show the demographic and clinical characteristics of the family caregivers and the patients, respectively.

**Table 1:** The demographic and clinical characteristics of family caregivers

Family care givers		
*n*=37		
	mean	SD
Age,years	58.6	15.3
Sex	n	%
Male	15	41
Female	22	59
Relationship	n	%
Husband	10	27
Wife	10	27
Daughter	10	27
Mother	1	3
Sister	2	5
Sibling	4	11
J-ZBI	mean	SD
	16.8	11.5

*J-ZBI* Japanese version of Zarit Caregiver Burden Interview

**Table 2:** The demographic and clinical characteristics of patients

Patients		
*n*=37		
	mean	SD
Age,years	66.9	10.7
Sex	n	%
Male	8	22
Female	29	78
ECOG PS	n	%
2	9	24
3	17	46
4	11	30
Primary cancer site	n	%
Brest	4	11
Gastrointestinal	13	35
Lung	2	5
Liver , Pancteas,biliary,system	6	16
Gynecomogical	6	16
Urological	1	3
Head and neck	5	14
Condition of the patients with advanced cancer	n	%
Under chemotherapy	21	57
Best supportive care	16	43

*ECOG PS* Eastern Cooperative Oncology Group Performance status

In this study, the mean HRV for 5 min before holding a hand or staying beside the patient was used as the baseline value.

Table 3 shows the mean HRV before and during the Handholding and Beside interventions and the comparison of HRV using two-way ANOVA in family caregivers. In a comparison of changes in HRV before and during intervention analyzed using a paired t-test, SDNN and LF score were significantly increased in the Handholding trial (SDNN, *p* = 0.022; LF, *p* = 0.036) and significantly decreased in the Beside trial (SDNN, *p* = 0.010; LF, *p* = 0.026). In terms of HF, no significant changes were found in either of the trials (Handholding, *p* = 0.893; Beside, *p* = 0.087).

**Table 3:** Change in the heart rate variability of family caregivers

Familygroup											
*n*= 37											
	Hand holding		Beside		Maineffect of Time course		Main effect of Trial		Interaction Effect		BonferroniPostHocTests
Characteristic	mean	SD	mean	SD	Fscore	pvalue	Fscore	pvalue	Fscore	pvalue	Trial
SDNN											
Before	33.3	(15.5)	37.1	(18.0)	0.177	0.674	2.088	0.151	7.669	0.006	*p*=0.351
During	42.6	(22.5)	30.3	(15.7)							*p*=0.003
LF											
Before	544.1	(530.9)	738.0	(745.3)	0.384	0.536	0.013	0.910	4.917	0.028	*p*=0.128
During	765.6	(858.7)	463.3	(410.0)							*p*=0.111
HF											
Before	521	(570.3)	456.1	(544.7)	0.391	0.533	2.348	0.128	0.599	0.440	
During	533.7	(568.6)	336.5	(327.5)							

*SDNN* standard deviation of inter beat interval, *LF* low frequency, *HF* High frequency

Table 4 shows the mean HRV before and during the Handholding and Beside interventions and the comparison of HRV using two-way ANOVA in patients. In a comparison of changes in HRV before and during intervention analyzed using a paired t-test, SDNN and LF increased significantly during the intervention in both trials (Handholding trial: SDNN, *p* = 0.002, LF, *p* = 0.014; Beside trial: SDNN, *p* = 0.049, LF, *p* = 0.012). In terms of HF, no significant changes were found in either of the trials (Handholding, *p* = 0.055; Beside, *p* = 0.114).

**Table 4:** Change in the heart rate variability of patients

Patientsgroup										
*n*= 37										
	Hand holding		Beside		Maineffect of Time course		Main effect of Trial		Interaction Effect	
Characteristic	mean	SD	mean	SD	Fscore	pvalue	Fscore	pvalue	Fscore	pvalue
SDNN										
Before	24.7	(13.6)	26.0	(11.8)	6.254	0.014	0.009	0.924	0.331	0.566
During	32.7	(19.0)	30.9	(16.4)						
LF										
Before	309.4	(465.1)	253.2	(264.7)	5.729	0.018	0.026	0.872	0.367	0.545
During	440.0	(511.9)	472.6	(472.6)						
HF										
Before	300.2	(470.0)	244.0	(453.6)	3.355	0.069	0.270	0.604	0.036	0.850
During	430.4	(482.8)	404.2	(495.3)						

*SDNN* standard deviation of inter beat interval, *LF* low frequency, *HF* High frequency

## Discussion

This is the first study that evaluated the effect of handholding on HRV of both patients and their family caregivers.

All patients had incurable cancer, and their HRV was lower than the average HRV of healthy adults [18]. Additionally, the median PS of the patients was 3, which indicates that the patients were capable of only limited self-care and spent more than 50 % of their waking hours in bed or in a chair [19]. A previous study indicated that poor patient health is associated with family caregivers’ sense of guilt, which hinders them from practicing self-care [1]. The family caregivers in this study were also in a situation where they were aware of the patient’s worsened condition and were less likely to practice self-care.

Our results provide two important perspectives. First, handholding can improve the HRV of family caregivers. To date, no studies have examined the effects of handholding on family caregivers. Because family caregivers feel guilty about spending time engaging in self-care [1], it is significant that daily handholding can become a form of care for themselves. Previous studies examined resonant breathing interventions [7] and music interventions with nursing presence [20] as direct interventions to improve the HRV of family caregivers of cancer patients. A significant increase in SDNN and LF was observed with the resonant breathing intervention, and a significant decrease in the LF/HF ratio, which is an indicator of sympathetic nerve activity, was observed in music interventions in the presence of nurses. However, these methods require special resources and the family caregiver’s time, which can be challenging for continuous care and widespread use.

The mechanism underlying the effects of handholding on autonomic nervous activity may be explained as follows: the stimulus of holding a hand is projected to the somatosensory area of the cerebral cortex via the brain stem reticular formation through the cutaneous sensation and the interoceptors of the muscle spindle [21]. Then, the hypothalamus stimulates the internal organs including the heart and lungs via the autonomic nervous system, causing fluctuations in heart rate [22–24].

The second important finding is that the patient’s HRV improves when family caregiver holds their hand or just sits beside them. A previous study showed that family caregivers feel less guilty about self-care if they have a meaningful relationship with the patient [1]. In fact, after the family caregivers received feedback that handholding increased the patient’s gastric motor function, their sense of guilt for self-care decreased from a numerical rating scale (NRS) of 7.0 to 4.9, and their motivation for self-care increased from an NRS of 3.3 to 6.4 [13]. If the action taken by the family caregiver has a positive effect on the patient and the patient’s change improves the family caregiver’s sense of self-care, this action is considered an indirect intervention of self-care support for the family caregiver. In this study, the increase in the patient’s HRV may have led to indirect self-care for the family caregiver, but the effect could not be evaluated because the family caregivers did not receive feedback on the changes in the patients.

In previous studies that evaluated gastric motor function, the motility index and gastric emptying rate increased significantly while the family caregiver was holding the patient’s hand [12, 13]. Evidence-based approaches have not been established because actions like touching are associated with multiple factors [25]. Although the results of this study suggest that the presence of a family caregiver beside the patient may have a significant impact on the patient’s HRV, further research is required.

In this study, HF showed no significant difference in the time course of the Handholding and Beside trials in both the patient and family caregiver groups. Moreover, no interaction was observed in either the patient or family caregiver group. The reason for this may be that the efferent pathway in the neural mechanism of somatic internal reflexes through the skin of the extremities is the sympathetic nerve [26]. This also indicates that the changes in SDNN and LF in this study may be associated with sympathetic nerves.

The present study has several limitations: (1) there was a participant bias because those who were comfortable with handholding were more likely to participate in this study; (2) the patients may have had diseases known to affect autonomic function such as diabetes, but this was unable to be determined because medical history was not included in the exclusion criteria; (3) there is a generalization problem because this study was conducted in a single facility; (4) the relationship between patients and caregivers, such as family intimacy and family-historical events, might be partly associated with the autonomic function; (5) a large variation was observed in HRV. Larger scale data are required in future studies to eliminate the effects of variability within subgroups and increase reliability.

## Conclusions

Handholding improves the autonomic function of family caregivers and may work as a form of self-care for family caregivers.

## Data Availability

The datasets analyzed in the current study are available from the corresponding author upon reasonable request.

## Abbreviations

HRV: Heart rate variability
SDNN: Standard deviation of the normal-to-normal interval
J-ZBI: Japanese version of Zarit Caregiver Burden Interview
ECOG: Eastern Cooperative Oncology Group
PS: Performance status
LF: Low frequency
HF: High frequency
NRS: Numerical rating scale
